# Promoting Physical Activity in Pediatric Oncology. Where Do We Go from Here?

**DOI:** 10.3389/fonc.2013.00173

**Published:** 2013-07-12

**Authors:** Carolina Chamorro Viña, Amanda J. Wurz, S. Nicole Culos-Reed

**Affiliations:** ^1^Faculty of Kinesiology, University of CalgaryCalgary, AB, Canada; ^2^Department of Psychosocial Resources, Tom Baker Cancer CentreCalgary, AB, Canada; ^3^Department of Oncology, Faculty of Medicine, University of CalgaryCalgary, AB, Canada

## Introduction

Advances in treatment and technology over the last 30 years are credited with the improved survival rates (in excess of 80%) in pediatric oncology (1). This growing survivor population has resulted in an increased awareness among clinicians about the negative side effects that may develop after treatments (1, 2). Physical side effects may include diminished muscular strength, peripheral neuropathy, decreased functional capacity, and increased fatigue (3–6). Psychosocial side effects may include elevated levels of fear and anxiety, poor social functioning, and decreased health related quality of life (HRQoL). These physical and psychosocial side effects are well-documented consequences of childhood cancer and occur in at least half of the population (4, 5, 7–10). These common physical and psychosocial side effects, in combination with a sedentary lifestyle, may further aggravate and accelerate the development of physical inactivity-related diseases such as hypertension, diabetes, coronary artery disease, osteoporosis, and cancer recurrence (11–13). The Childhood Cancer Survivor Study found that 62% of childhood cancer survivors had at least one chronic condition and 27.5% had a severe, life-threatening, or disabling condition (1). Recently, physical activity (PA) intervention research has emerged as a promising adjuvant therapy to mitigate the negative effects of cancer and its treatment. In this opinion article: (1) the rationale for PA in pediatric cancer (patients and survivors) will be summarized, (2) the challenges to promoting PA behaviors in this population will be discussed, and (3) our vision for the next steps (research, education, and knowledge translation) will be highlighted.

## Evidence for PA in Pediatric Cancer

There is growing evidence for the efficacy of PA to improve strength, cardiorespiratory fitness, symptoms of fatigue, and general physical functioning in pediatric cancer populations (11, 13–16). While the effect of PA on HRQoL outcomes, such as functional, social, and mental domains is mixed, the majority of the research suggests potential improvements (15). Additionally, none of the studies to date have reported any negative outcomes, even in immune-compromised patients (17).

Due to the relative rarity of a pediatric cancer diagnosis (153 cases/per million children in Canada), evaluating the effect of PA in this population is inherently more challenging (13). Although findings are preliminary, and are often limited by small sample sizes, the existing evidence consistently suggests that mild-moderate exercise is safe, beneficial, and feasible for both childhood patients and survivors (11).

## Sedentary Behavior in Pediatric Cancer

Scientific evidence suggests that childhood cancer survivors are more sedentary than their healthy peers (14, 18, 19). Three potential explanations have emerged in the literature (20). The first, a physiological explanation, postulates that survivors of cancer are less active because they experience fatigue as a result of the adverse consequences of chemotherapy and radiotherapy (21, 22). The second argues that childhood cancer survivors are subject to an insidious “spectrum of disuse” as a result of an overly cautious approach toward PA fostered by concerned parents, physicians, school teachers, and others (23). This overprotective attitude may stem from a general lack of education about what a child can be expected to do after cancer diagnosis and treatments, and may alter the child's perception of their actual capacity for PA (20). As an example, more than 70% of school physical education teachers reported a lack of knowledge about appropriate activities for childhood cancer survivors (24). The third explanation suggests that the timing of the diagnosis and the length of the treatment often coincide with a period of life when children are introduced to organized sports, resulting in the child missing out on this critical period and not being introduced to numerous PA options (15, 25).

## The Importance of Physical Fitness and PA during Childhood

Physical activity is one of the main determinants of physical fitness (26) and has been suggested to improve cardiovascular capacity, strength, HRQoL, and daily functioning in a wide range of pediatric chronic diseases (i.e., juvenile idiopathic arthritis, asthma, cerebral palsy, and cystic fibrosis) (9, 27, 28). In healthy populations, research shows that physical inactivity is an independent risk factor for non-communicable diseases (NCD) such as obesity, diabetes, hypertension, cardiovascular disease, and cancer (29, 30). Recognizing and intervening on physical inactivity during childhood is an important public health concern. Enhancing PA during childhood is necessary to: (a) prevent and treat NCD, (29, 30), (b) promote the adoption of PA behavior that will translate into adulthood (29), and (c) improve aerobic capacity and muscle mass and maximize peak bone mineral density (31–33).

## How Do We Promote PA in Pediatric Cancer?

Physical activity is a key factor in the development of healthy children. Due to the high levels of sedentary behavior reported in children, a recent article proposed integrating exercise assessments into primary care as a means of intervening before a childhood exercise deficit disorder occurs (34). The importance of intervening in childhood cancer patients and survivors is necessary, as they are at a greater risk (compared to healthy children) to develop a sedentary lifestyle and the associated co-morbid conditions. Due to the varying hypotheses for physical inactivity in this population, it is clear that several factors must be taken into account when developing strategies to improve the PA participation rates of childhood cancer patients and survivors. We acknowledge that continued research is necessary to develop adequately powered, solidly designed PA research interventions to continue exploring key issues within pediatric oncology (15, 35). However, the evidence to date consistently suggests that mild-moderate PA is safe, beneficial, and feasible in this population (13–15, 35). Therefore, enhancing the dissemination of the evidence that already exists is an important next step.

### Developing guidelines

In 2010, The American College of Sports Medicine published a roundtable report that brought together a team of clinical and research experts in adult cancer and exercise. The group advised “avoiding inactivity, even in cancer patients with existing disease or undergoing difficult treatments, is likely helpful” (36). While the pediatric literature is not as extensive, the evidence to date suggests we should promote an active lifestyle throughout the pediatric cancer experience in order to diminish many of the negative physical and psychosocial side effects (35). As a starting point, the Children's Oncology Group published The Long-Term Follow-Up Guidelines for survivors of childhood, adolescent, and young adult cancers (35). This guideline does not include any specific recommendations for PA, but does include a section on healthy living. Additionally, The American Cancer Society currently recommends 60 min of moderate-vigorous exercise 5 days/week in child and adolescent in order to prevent cancer (37). While specific PA guidelines cannot be developed due to the current state of the literature, other avenues may be used in order to increase community and health care professional (HCP) awareness regarding the benefits of exercise during childhood cancer.

Enhanced knowledge translation may begin to diminish the overly cautious approach of parents, physicians, and teachers, which has been acknowledged as one of the limiting factors for PA involvement (23). HCPs are a key factor in the promotion of PA in pediatric cancer. Survivors report that they are more willing to participate in PA if their HCP recommends it (14). Therefore, a practical evidence-based PA guideline that summarizes the evidence we already have, highlighting the strengths and limitations of the research, could give HCPs the tools to talk about exercise with their patients and appropriately refer to exercise experts when necessary. Furthermore, creating an exercise manual for a lay-audience may improve the inclusion of childhood cancer survivors in regular PA at school and at home. Increasing accessibility of the evidence would begin to address the lack of knowledge reported by parents, physicians, and teachers (24). Our research group is currently developing a Pediatric Oncology Exercise Manual (POEM) with an international team of researchers [Spain, Germany, Canada, Netherlands, and the United States of America (USA)]. POEM will summarize the pediatric cancer and exercise evidence that we have and make it more accessible for professionals and families. This manual will be given along with educational sessions for families and professionals.

### Knowledge translation: Creating and promoting evidence-informed exercise programs

Physical activity programing for pediatric cancer patients and survivors is growing. Worldwide, we have identified three programs based in the community. Pediatric Survivors Engaging in Exercise for Recovery (PEER) is offered for pediatric cancer patients and survivors at the University of Calgary. Play Strong – A Pediatric Cancer Exercise Program, was developed in the Nationwide Children's Hospital in Ohio, USA. The third was developed at the University of South Australia's School of Health Sciences in partnership with the Little Heroes Foundation Child Family Care Project and Leukemia Foundation. These three program have as part of theirs goals the restoration of healthy levels of physical fitness as well as provide childhood cancer survivors an opportunity to develop sports-based skills that allow them to return to PA classes designed for healthy children.

As the number of survivors continues to grow, expanding, and researching PA programs specifically for children with cancer should be a priority. PA research needs to occur in order to resolve the barriers that cancer and its treatment create, as well as assist with the reintegration of children back into the community (2, 13, 16).

Based on research evidence and what we have learned from our program, we suggest that community-based programs take into account: (a) medical history, (b) specialist supervision, (c) the fun factor, (d) parent and child education, and (e) frameworks/evaluation:

(a) Childhood cancer patients and survivors should be screened and cleared by their physician prior to participation in PA. It is imperative that an oncologist or primary care physician evaluates each patient based on diagnosis, therapy, long-term complications, and side effects (14, 38). This information can be given to exercise specialist, allowing them to tailor the exercise to each patient's medical history (14). Fluent communication with the patient's oncology team should occur to ensure the continued tailoring of the exercise sessions to individual's needs (13, 36).
(b) Programs should be supervised by appropriately trained individuals such as cancer and exercise specialists or certified exercise physiologists (14).
(c) The program should be fun while addressing psychological and pedagogical developmental needs. This component is a key factor to improve program adherence (2).
(d) Program should include educational component. Educating families and children on the benefits of PA may mitigate the overprotective attitudes parents hold and may help motivate children to perform PA (24).
(e) Program designs and evaluation should be based on theoretical frameworks. This will likely increase program success as well as the ability to adequately evaluate the program.

## Conclusion

There is a need to enhance awareness among HCP's, families, and educators about the benefits of PA throughout the pediatric cancer experience (diagnosis to survivorship). Evidence-based recommendations accessible to families, educators, and HCP's are needed and accessible programing's built upon this are the first steps to promoting better fitness and health in pediatric cancer patients and survivors. Fostering healthy PA habits and providing the infrastructure in which these habits can be developed will ultimately promote a healthy childhood cancer survivor population.

## References

[1] Oeffinger KC Mertens AC Sklar CA Kawashima T Hudson MM Meadows AT Chronic health conditions in adult survivors of childhood cancer. N Engl J Med (2006) 355(15):1572–82 10.1056/NEJMsa060185 17035650

[2] Takken T van der Torre P Zwerink M Hulzebos EH Bierings M Helders PJ Development, feasibility and efficacy of a community-based exercise training program in pediatric cancer survivors. Psychooncology (2009) 18(4):440–8 10.1002/pon.1484 19242926

[3] San Juan AF Chamorro-Vina C Mate-Munoz JL Fernandez del Valle M Cardona C Hernandez M Functional capacity of children with leukemia. Int J Sports Med (2008) 29(2):163–7 10.1055/s-2007-964908 17879894

[4] San Juan AF Fleck SJ Chamorro-Vina C Mate-Munoz JL Moral S Perez M Effects of an intrahospital exercise program intervention for children with leukemia. Med Sci Sports Exerc (2007) 39(1):13–21 10.1249/01.mss.0000240326.54147.fc 17218878

[5] Gocha Marchese V Chiarello LA Lange BJ Strength and functional mobility in children with acute lymphoblastic leukemia. Med Pediatr Oncol (2003) 40(4):230–2 10.1002/mpo.10266 12555250

[6] van Brussel M Takken T van der Net J Engelbert RH Bierings M Schoenmakers MA Physical function and fitness in long-term survivors of childhood leukaemia. Pediatr Rehabil (2006) 9(3):267–74 17050404 10.1080/13638490500523150

[7] Norris JM Moules NJ Pelletier G Culos-Reed SN Families of young pediatric cancer survivors: a cross-sectional survey examining physical activity behavior and health-related quality of life. J Pediatr Oncol Nurs (2010) 27(4):196–208 10.1177/1043454209358411 20173080

[8] Braam KI van der Torre P Takken T Veening MA van Dulmen-den Broeder E Kaspers GJ Physical exercise training interventions for children and young adults during and after treatment for childhood cancer. Cochrane Database Syst Rev (2013) 4:CD008796 10.1002/14651858.CD008796.pub2 23633361

[9] van Brussel M van der Net J Hulzebos E Helders PJ Takken T The Utrecht approach to exercise in chronic childhood conditions: the decade in review. Pediatr Phys Ther (2011) 23(1):2–14 10.1097/PEP.0b013e318208cb22 21304338

[10] San Juan AF Chamorro-Vina C Moral S Fernandez del Valle M Madero L Ramirez M Benefits of intrahospital exercise training after pediatric bone marrow transplantation. Int J Sports Med (2008) 29(5):439–46 10.1055/s-2007-965571 17960520

[11] Wolin KY Ruiz JR Tuchman H Lucia A Exercise in adult and pediatric hematological cancer survivors: an intervention review. Leukemia (2010) 24(6):1113–20 10.1038/leu.2010.54 20410923

[12] Finnegan L Wilkie DJ Wilbur J Campbell RT Zong S Katula S Correlates of physical activity in young adult survivors of childhood cancers. Oncol Nurs Forum (2007) 34(5):E60–9 10.1188/07.ONF.E60-E69 17878118

[13] San Juan AF Wolin K Lucia A Physical activity and pediatric cancer survivorship. Recent Results Cancer Res (2011) 186:319–47 10.1007/978-3-642-04231-7_14 21113771

[14] Kelly AK Physical activity prescription for childhood cancer survivors. Curr Sports Med Rep (2011) 10(6):352–9 10.1249/JSR.0b013e318237be40 22071396

[15] Huang TT Ness KK Exercise interventions in children with cancer: a review. Int J Pediatr (2011) 2011:461512 10.1155/2011/461512 22121378 PMC3205744

[16] Keats MR Culos-Reed SN A community-based physical activity program for adolescents with cancer (project TREK): program feasibility and preliminary findings. J Pediatr Hematol Oncol (2008) 30(4):272–80 10.1097/MPH.0b013e318162c476 18391695

[17] Chamorro-Vina C Ruiz JR Santana-Sosa E Gonzalez Vicent M Madero L Perez M Exercise during hematopoietic stem cell transplant hospitalization in children. Med Sci Sports Exerc (2010) 42(6):1045–53 10.1249/MSS.0b013e3181c4dac1 19997035

[18] Ness KK Leisenring WM Huang S Hudson MM Gurney JG Whelan K Predictors of inactive lifestyle among adult survivors of childhood cancer: a report from the childhood cancer survivor study. Cancer (2009) 115(9):1984–94 10.1002/cncr.24209 19224548 PMC2692052

[19] Ness KK Hudson MM Ginsberg JP Nagarajan R Kaste SC Marina N Physical performance limitations in the childhood cancer survivor study cohort. J Clin Oncol (2009) 27(14):2382–9 10.1200/JCO.2008.21.1482 19332713 PMC2738647

[20] Braith RW Role of exercise in rehabilitation of cancer survivors. Pediatr Blood Cancer (2005) 44(7):595–9 10.1002/pbc.20354 15747335

[21] Lucia A Earnest C Perez M Cancer-related fatigue: can exercise physiology assist oncologists? Lancet Oncol (2003) 4(10):616–25 10.1016/S1470-2045(03)01221-X 14554239

[22] Arroyave WD Clipp EC Miller PE Jones LW Ward DS Bonner MJ Childhood cancer survivors’ perceived barriers to improving exercise and dietary behaviors. Oncol Nurs Forum (2008) 35(1):121–30 10.1188/08.ONF.121-130 18192161

[23] Aznar S Webster AL San Juan AF Chamorro-Vina C Mate-Munoz JL Moral S Physical activity during treatment in children with leukemia: a pilot study. Appl Physiol Nutr Metab (2006) 31(4):407–13 10.1139/h06-014 16900230

[24] Robertson AR Johnson DA Rehabilitation and development after childhood cancer: can the need for physical exercise be met? Pediatr Rehabil (2002) 5(4):235–40 12745903 10.1080/1363849031000094072

[25] Oeffinger KC Buchanan GR Eshelman DA Denke MA Andrews TC Germak JA Cardiovascular risk factors in young adult survivors of childhood acute lymphoblastic leukemia. J Pediatr Hematol Oncol (2001) 23(7):424–30 10.1097/00043426-200110000-00007 11878576

[26] Ortega FB Ruiz JR Castillo MJ Sjostrom M Physical fitness in childhood and adolescence: a powerful marker of health. Int J Obes (2008) 32(1):1–11 10.1038/sj.ijo.0803774 18043605

[27] Rand S Prasad SA Exercise as part of a cystic fibrosis therapeutic routine. Expert Rev Respir Med (2012) 6(3):341–51 10.1586/ers.12.19quiz352 22788948

[28] Crosbie A The effect of physical training in children with asthma on pulmonary function, aerobic capacity and health-related quality of life: a systematic review of randomized control trials. Pediatr Exerc Sci (2012) 24(3):472–89 22971562 10.1123/pes.24.3.472

[29] Swaminathan S Vaz M Childhood physical activity, sports and exercise and noncommunicable disease: a special focus on India. Indian J Pediatr (2012) 80:S63–70 10.1007/s12098-012-0846-1 22791355

[30] Mendes R Sousa N Barata JL Physical activity and public health: recommendations for exercise prescription. Acta Med Port (2011) 24(6):1025–30 22713198

[31] Faigenbaum AD Kraemer WJ Blimkie CJ Jeffreys I Micheli LJ Nitka M Youth resistance training: updated position statement paper from the national strength and conditioning association. J Strength Cond Res (2009) 23(5 Suppl):S60–79 10.1519/JSC.0b013e31819df407 19620931

[32] Fleg JL Aerobic exercise in the elderly: a key to successful aging. Discov Med (2012) 13(70):223–8 22463798

[33] Pitukcheewanont P Punyasavatsut N Feuille M Physical activity and bone health in children and adolescents. Pediatr Endocrinol Rev (2010) 7(3):275–82 20526241

[34] Stracciolini A Myer GD Faigenbaum AD Exercise-deficit disorder in children: are we ready to make this diagnosis? Phys Sportsmed (2013) 41(1):94–101 10.3810/psm.2013.02.2003 23445864

[35] Zhang FF Saltzman E Must A Parsons SK Do childhood cancer survivors meet the diet and physical activity guidelines? A review of guidelines and literature. Int J Child Health Nutr (2012) 1:44–58 10.6000/1929-4247.2012.01.01.06 PMC4786177 26973721

[36] Schmitz KH Courneya KS Matthews C Demark-Wahnefried W Galvao DA Pinto BM American college of sports medicine roundtable on exercise guidelines for cancer survivors. Med Sci Sports Exerc (2010) 42(7):1409–26 10.1249/MSS.0b013e3181e0c112 20559064

[37] Doyle C Kushi LH Byers T Courneya KS Demark-Wahnefried W Grant B Nutrition and physical activity during and after cancer treatment: an American cancer society guide for informed choices. CA Cancer J Clin (2006) 56(6):323–53 10.3322/canjclin.56.6.323 17135691

[38] Oeffinger KC Hudson MM Landier W Survivorship: childhood cancer survivors. Prim Care (2009) 36(4):743–80 10.1016/j.pop.2009.07.007 19913185

